# Simulations of a full sonoreactor accounting for cavitation

**DOI:** 10.1016/j.ultsonch.2022.106226

**Published:** 2022-11-11

**Authors:** Igor Garcia-Vargas, Laurie Barthe, Pascal Tierce, Olivier Louisnard

**Affiliations:** a Centre RAPSODEE, IMT Mines-Albi, UMR CNRS 5302, Université de Toulouse, 81013 Albi CT, France; b Laboratoire de Génie Chimique, Université de Toulouse, CNRS, INPT, UPS, Toulouse, France; c SinapTec, 7, Avenue Pierre et Marie Curie, 59260 Lezennes, France

**Keywords:** Acoustic cavitation, Simulation, Piezoelectric transducers, Ultrasound propagation, Attenuation

## Abstract

In spite of the increasing interest in ultrasound processing applications, industrial scale-up remains limited, in particular by the unavailability of predictive computer tools. In this study, using a previously published model of cavitating liquids implementable as a non-linear Helmholtz equation, it is shown that a full sonoreactor can be modelled and simulated. The model includes the full transducer and the vibrations of the vessel walls, using the physics of elastic solids and piezo-electricity. The control-loop used by the generator to set the optimal frequency is also accounted for. Apart from the geometry, the unique input of the model is the current feeding the transducer whereas the dissipated electrical power, transducer complex impedance and working frequency are available as outputs. The model is put to the test against experiments realized in different geometries, varying either the input current or the transducer immersion depth. Despite the overestimation of the power dissipated in the liquid, the evolution of the acoustic load in both cases is reasonably well reproduced by simulation, which partially validates the method used.

## Highlights

• A full sonoreactor, transducer/cavitating liquid/vessel walls, is simulated.
• The US generator’s automatic frequency selection is accounted for.
• The current feeding the transducer is the unique model input.
• The dissipated electrical power is compared to experiments.
• Transducer immersion depth strongly influences the power dissipated.

## 1 Introduction

When a liquid is irradiated with intense ultrasound, thousands of gas micro-bubbles appear and collapse violently, leading to the release of an enormous amount of energy in the bubbles. This phenomenon known as acoustic cavitation [1] induces drastic physical, mechanical and chemical effects and is used in various industrial applications, such as ultrasonic cleaning [2], [3], wastewater treatment [4], [5], [6], extraction [7], [8], emulsification [9], food processing [10], [11], [12], polymerization [13], atomization [14], hydrogen production [15], [16] and sonochemistry [17], [18], [19], [20], [21]. The development of stabilized coated microbubbles has also triggered various medical applications [22], [23], [24].

In the 1980s and 1990s, there was a rapid resurgence of interest in cavitation and sonochemistry applications, mainly at the laboratory scale. As any growing science, the difficulty its extrapolation to the industrial scale inevitably occurred, and raised critical issues. The phenomena occurring within a sonoreactor are very complex, and unfortunately, unlike more traditional branches of process engineering, there is currently hardly any tool to design sonochemical reactors. One reason for this is the great physical complexity of acoustic cavitation. The enormous range of spatial and temporal scales involved, for example, makes dimensional analysis impractical. On the other hand, because ultrasound is employed, acoustics should be one of the most significant ingredients of sonoreactors science. Nevertheless, it remains one of the most disregarded in prior research.

To shed new light on this issue, several groups have attempted to simulate the acoustic field inside sonoreactors in order to predict cavitation events within the reactor see [25] for a review.

Most simulations rely on linear acoustic [26], [27], [28], [29], [30], [31], [32], [33], [34], [35], [36], [37], [38], [39], [40], which takes the form of an Helmholtz equation and can be easily solved by the finite-elements method (FEM). The main drawback of such an approach is that the effect of cavitation on the acoustic field is not accounted for, apart for some studies using an empirical attenuation coefficient. In order to account for the presence of bubbles, nonlinear models of bubbly liquids acoustics should be used [41], [42], [43], but the latter are difficult to solve on large spatial scales, despite interesting results have been obtained on reduced scales by using involved numerical methods [44], [45]. Several groups [46], [47], [48], [49], [50], [51], [52], [53], [54] have used the linearized version of the Caflisch model developed by Commander & Prosperetti [55]. As the latter assumes linear oscillations of the bubbles, this approach yields unrealistic values of the attenuation coefficient where inertial cavitation is involved [56] (see also Ref. [57] for an in-depth discussion).

Bubbles are the main dissipators in the liquid. Each bubble dissipates mechanical energy into heat and the sum of these contributions is the power measured by the calorimetric method. The above-mentioned studies cannot therefore compute correctly the calorimetric power, either because bubbles are neglected, or because the energy they dissipate is largely underestimated by linearized bubble models [58]. To get rid of the latter limitation, Louisnard [59], [60] developed a reduced non-linear model of sound propagation in cavitating liquids, based on Caflisch’s model and accounting for fully nonlinear bubble dynamics. As the proposed nonlinear Helmholtz equation directly involves the power dissipated by each bubble, computing the power dissipated in the liquid becomes natural within this model. This model has been successfully used to predict commonly observed cavitation phenomena [60], such as conical bubble structures under ultrasonic horns [61] or flare structures that are very similar to those observed experimentally in ultrasonic cleaning tanks [62]. Several refinements have been proposed since then, to account for radiation losses [63], [64]. Sojahrood and co-workers have extended the theory in various aspects [65], including extension to coated microbubbles [66], [67]. The latter group also recently performed experiments with a layer of almost monodisperse coated microbubbles to assess experimentally the attenuation and sound-velocity in the bubbly liquid and found good agreement between their extension of our model and the experimental results [68]. Both attenuation and sound velocity were found to vary with acoustic pressure, and to our best knowledge, this constitutes the first experimental results supporting the concepts raised by Louisnard in Ref. [59]. Other groups have recently used Louisnard’s model for different fluids other than water [69], in liquid aluminium [70], at frequencies larger than 20kHz [71]. Interesting results have been obtained by Lesnik and co-workers, who coupled our model with the equations governing the translational motion of bubbles and their effect on the fluid motion [72].

A sonoreactor consists of a volume of liquid enclosed within solid walls, in contact with one (or more) transducer, which in turn is driven by an electrical generator. Using the reasonably realistic model for the cavitating liquid of Ref. [59], simulating the full assembly consists in modelling the transducer and coupling its vibrations with the one of the liquid. Modelling transducers has now become an easy task thanks to the implementation of the piezo-electricity equations in most FEM codes, in our case COMSOL Multiphysics. Moreover, the equations of mechanical coupling between the solid vibrations and the acoustics of the liquid are classical [73] and can also be used to study the recipient wall vibrations [31], [57]. Therefore, as far as the internal design of the transducer is precisely known, and circumventing the additional difficulty brought by the automatic frequency selection implemented on modern ultrasound generator, modelling a full sono-reactor under COMSOL becomes feasible. Demonstrating this assertion is the first objective of this paper.

In order to test our numerical predictions, we also performed a significantly large set of experiments by varying several controllable parameters: the input current amplitude, which is the quantity experimentally controlled by the generator level button, the geometry of the enclosing vessel, and the immersion depth of the transducer.

The paper is organized as follows: Section 2 describes the experimental setup, the set of experiments realized and the principles underlying the frequency control loop of the generator. Section 3 describes the model. We first recall the main lines of Louisnard’s model [59], give a summary of the equations used for solid vibrations, including piezo-electrics, and how we account for the automatic frequency control. Section 4 shows the comparison between numerical and experimental results, for the two set of experiments, varied current and varied immersion depth. The agreement between the simulated and experimental results is discussed in Section 5, along with suggested future enhancements of Louisnard’s model.

## 2 Experimental

### 2.1 Experimental setup

**Fig. 1:**
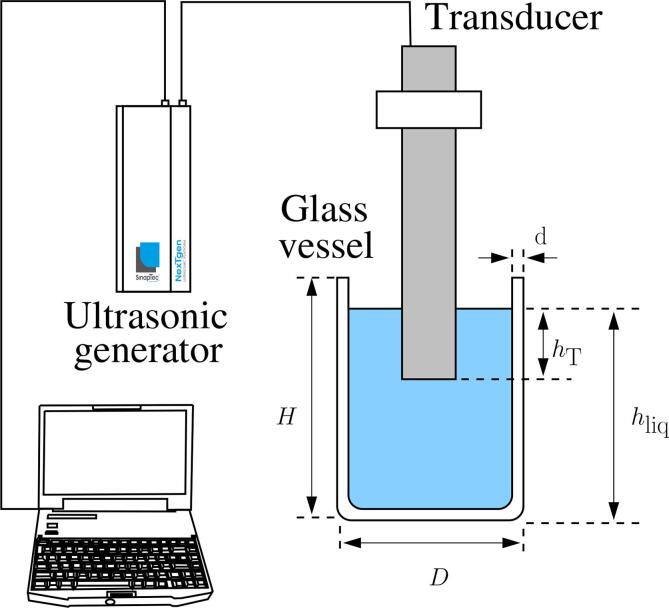
Schematic of the experimental setup.

A schematic of the experimental setup is shown in Fig. 1. The apparatus used in this study was a 20kHz homemade standard Langevin-type transducer (SinapTec, Lezennes, France) with two piezoelectric rings pre-stressed by a steel screw between a mass and counter-mass, both made of titanium alloy. The exact resonance frequency of the unloaded transducer, $f_{0}$ = 20342Hz, was measured with an impedance-bridge. The transducer was driven by a computer-controlled ultrasonic generator (SinapTec NexTgen Inside 500), by which several electrical quantities, such as power, frequency, voltage and current and their mutual phase, impedance etc, can be monitored and logged every 50ms during experiments.

### 2.2 Sets of experiments

The transducer was immersed in the center of three different commercial glass beakers (hereinafter referred to as A, B and C) filled with tap water. The dimensions of the beakers are shown in Table 1.

**Table 1:** Geometrical characteristics of the beakers (see Fig. 1).

Beaker	Form	Capacity [L]	*D* [cm]	*H* [cm]	*d* [mm]
A	wide	1	10.5	14.3	2.3
B	narrow	2	11.95	23.7	2.5
C	wide	2	13.2	18.3	2.2

For each beaker, two additional geometrical parameters can be varied: the transducer immersion depth $h_{T}$ and the water level $h_{\mathrm{liq}}$ (Fig. 1). In this study, two sets of experiments were carried out. In the first set, both the transducer immersion depth $h_{T}$ and the liquid height $h_{\mathrm{liq}}$ were kept at constant values for each beaker, whereas the input current of the transducer was varied. For beaker A, the transducer immersion depth was arbitrarily fixed at $h_{T}=3\;\mathrm{cm}$ and the liquid height $h_{\mathrm{liq}}$ at 12 cm. For beakers B and C the values of $h_{T}$ and $h_{\mathrm{liq}}$ were chosen so that the two ratios $h_{\mathrm{liq}}/H$ and $h_{T}/H$ remain beaker independent. The corresponding values of $h_{T}$ and $h_{\mathrm{liq}}$ can be found in Table 2. The input RMS current provided by the generator was varied from 0.08 to 0.8 A in all experiments of set 1.

**Table 2:** Summary of parameters used in experiments.

Experiment set	Beaker	*H* [cm]	$h_{T}$ (cm)	$h_{\mathrm{liq}}$ (cm)	$I_{0,\mathrm{RMS}}$ (A)
	A	14.3	3.0	12.0	
Set 1	B	23.7	5.0	19.9	0.08 to 0.8
	C	18.3	3.8	15.3	
					
	A	14.3	0.5 to 10.0	Computed	
Set 2	B	23.7	1.8 to 12.0	from Eq. (1)	0.6
	C	18.3	1.7 to 12.2		

**Fig. 2:**
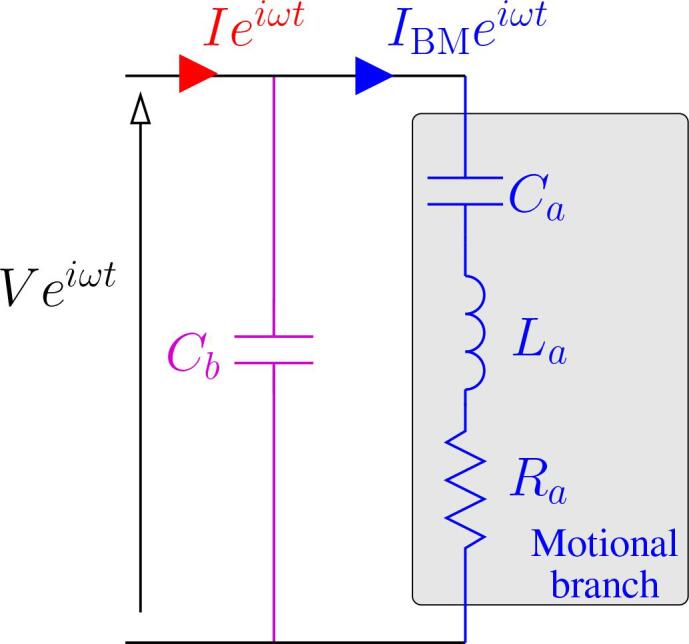
Equivalent circuit for the transducer [75]. The capacitor $C_{b}$ originates from the pure dielectric character of piezo-electrics. The motional branch reflects the mechanical load of the transducer.

In the second set of experiments, the current was fixed at a constant value of $I_{0,\mathrm{RMS}}=0.6\;A$ and $h_{T}$ was swept in a beaker-dependent range (Fig. 2). For each beaker, the liquid volume $V_{\mathrm{liq}}$ was kept constant so that the liquid level $h_{\mathrm{liq}}$ varied in function of immersion depth $h_{T}$ following:

(1) $$V_{\mathrm{liq}}=\pi \frac{D_{\mathrm{int}}^{2}}{4}h_{\mathrm{liq}}-\pi \frac{D_{T}^{2}}{4}h_{T}$$

where $D_{\mathrm{int}}=D-2d$ is the beaker internal diameter and $D_{T}$ is the transducer diameter.

Finally, for each set of current/ transducer depth, the liquid was irradiated continuously for only for 30s to avoid heating, at ambient temperature and pressure, in all experiments. As the generator internal control loop was able to set the frequency in less than 1s, all the electrical data monitored during the first two seconds were removed. The remaining data were averaged for each experiment, and the standard deviation of the fluctuations was calculated, yielding an error bar for each point. In summary, all the measured electrical quantities presented hereinafter correspond to averages/standard deviations over 28s with data points logged every 50ms.

### 2.3 Frequency control loop

When the transducer is immersed, it operates against a mechanical load and is therefore in contact with a non-zero complex mechanical impedance. This has the effect to slightly shift the resonance frequency and possibly results in a reduction of the vibration amplitude [74]. To circumvent this problem, ultrasonic generators usually employ some control strategy to ensure a constant vibration amplitude of the ultrasonic tool by adjusting in real time the frequency of the system to the load.

In the vicinity of the resonance frequency, the vibration behavior of the transducer can be described by the equivalent circuit displayed in Fig. 2 [75]. The capacitive branch $C_{b}$accounts for the purely dielectric properties of the piezo-electric rings, whereas the motional branch in blue (RLC circuit in series) reflects the mechanical properties of the vibrating element and the load. It is important to note that all the energy dissipation mechanisms are concentrated in the resistance $R_{a}=RZ_{\mathrm{BM}}$. The latter include energy dissipated into heat in the transducer, but more importantly, all the energy dissipated in the load (for example by cavitation). The branch $C_{b}$ is classically compensated by a parallel matching inductance so that only the motional branch remains at resonance. The control strategy of the generator consists in estimating the complex motional impedance $Z_{\mathrm{BM}}=V/I_{\mathrm{BM}}$ and to adjust the frequency so that its reactive part vanishes ($IZ_{\mathrm{BM}}=0$). This ensures that the whole available input power is transferred to the load, whatever the fluctuations of the latter. The additional control of the input current amplitude allows a good stability in operating conditions.

## 3 Numerical study

**Fig. 3:**
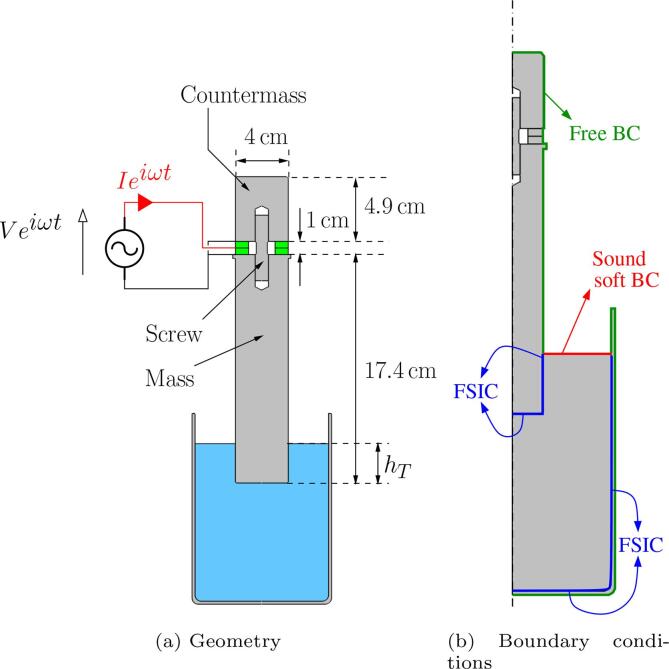
(a) Sketch of the geometry used in the simulation. The green rectangle connected to the generator are the piezo-electric rings. (b) Mechanical boundary conditions (BC) for the solid and acoustic boundary conditions for the liquid. The blue lines labelled as FSIC (Fluid Structure Interface Conditions) couple the liquid acoustics to the solid vibrations.

The model detailed hereinafter was implemented in the commercial code COMSOL Multiphysics, based on the Finite Element Method (FEM). The numerical modeling was performed in the 2D-axisymmetric domain and the sketch of the modelled setup is presented in Fig. 3a. Similar computation could be easily extended to 3D geometries if required, at the expense of heavier computational resources.

### 3.1 Cavitating liquid

In order to account for acoustic energy dissipated by cavitation, the liquid was modelled by a previously published reduced nonlinear model [59], based on the rigorously derived Caflisch equations [43] describing acoustics of bubbly fluids. Our model results in a nonlinear Helmholtz equation, whose squared wavenumber is expressed in function of the energy dissipated by cavitation bubbles by thermal diffusion and by viscous friction in the liquid along their radial oscillations around the bubble. The latter energies are estimated by solving a fully nonlinear bubble dynamics equation. The nonlinear Helmholtz equation is used only in zones where inertial cavitation takes place, i.e. where the acoustic pressure exceeds the Blake threshold. Assuming mono-harmonic oscillations and setting the pressure field as $p(r,t)=\frac{1}{2}P(r)e^{i\omega t}+c.c\text{.}$ (c.c. = complex conjugate), the equations of the model can be summarized as:

(2c) $$\nabla^{2}P+k_{l}^{2}P=0\;\mathrm{where}\;|P|<P_{B}\;(\mathrm{no}\;\mathrm{bubbles}),$$

(2d) $$\nabla^{2}P+k_{\mathrm{NL}}^{2}P=0\;\mathrm{where}\;|P|⩾P_{B}\;(\mathrm{bubbles}),$$

where $k_{l}=\omega /c_{l}$ , $P_{B}$ is the Blake threshold [76], [77], [78]:

(3) $$P_{B}=p_{0}\left(1+{\left(\frac{4}{27}\frac{S^{3}}{1+S}\right)}^{1/2}\right),$$

$S=2\sigma /(p_{0}R_{0})$ is the dimensionless Laplace tension. ω is the angular frequency, $c_{l}$ the sound velocity in pure liquid, $p_{0}$ the static pressure, σ the surface tension, $R_{0}$ bubble ambient radius. The nonlinear wavenumber $k_{\mathrm{NL}}$ is defined by:

(4) $$\text{Re}(k_{\mathrm{NL}}^{2})=\frac{\omega^{2}}{c_{l}^{2}}+\frac{4\pi {\mathrm{NR}}_{0}\omega^{2}}{\omega_{0}^{2}-\omega^{2}},$$

(5) $$\text{Im}(k_{\mathrm{NL}}^{2})=-2N\rho_{l}\omega \frac{\Pi_{v}(|P|)}{|P{|}^{2}},$$

where $\omega_{0}={\left[(3(1+S)-S)p_{0}/(\rho_{l}R_{0}^{2})\right]}^{1/2}$ is the bubble isothermal resonance frequency, and $\Pi_{v}(|P|)$ the viscous dissipation function. The latter is pre-computed by solving a Keller equation augmented with a reduced model accounting for the heat and water transfer between the bubble and the surrounding liquid (see A), fitted, and injected in COMSOL as an analytical function [59], [57]. The nonlinear Helmholtz equation is implemented within the *Pressure Acoustics* COMSOL physics module.

For all the simulation results presented hereinafter, we considered air bubbles of ambient radius $R_{0}=5\mu \text{m}$ in water at ambient temperature and pressure, and the bubble density was set to 50bubbles/mm^3^, unless otherwise specified. This choice is justified by experimental results which shows that at 20kHz, the ambient bubble sizes lie in a narrow range between 2μm and 10μm [79], [80]. This is moreover consistent with theoretical results on bubble shape instabilities leading to bubble breakup above some critical size [81], [82], [83], [84], [85], [86]. The order of magnitude of tens of bubbles per mm^3^ for *N* is mentioned in [62] and the selected value *N* = 50bubbles/mm^3^ yields void fractions $\beta =4/3\pi R_{0}^{3}N≃2.6\times 10^{-5}$, which is of the order of magnitude of results reported in Ref. [79].

The computational domain and the associated boundary conditions (BC) are illustrated in Fig. 3b. On the liquid surface, a soft boundary condition (P=0) was set. The conditions to be applied on the liquid boundaries in contact with solid parts are deferred in the next section.

### 3.2 Transducer and vessel wall

The motion of linear elastic materials (mass, screw, counter-mass, and vessel boundaries) can be represented by Newton’s equation complemented with Hooke’s law [31]. In time-harmonic form at frequency ω, the equations read:

(6) $$-\rho_{S}\omega^{2}U_{S}=\nabla \cdot \overset{‾}{\overset{‾}{T}},$$

(7) $$\overset{‾}{\overset{‾}{T}}=c\overset{‾}{\overset{‾}{S}},$$

(8) $$\overset{‾}{\overset{‾}{S}}=\frac{1}{2}\left(\overset{‾}{\overset{‾}{\nabla }}U_{S}{+}^{T}\overset{‾}{\overset{‾}{\nabla }}U_{S}\right),$$

where $U_{S}$ is the mono-harmonic displacement field amplitude, $\overset{‾}{\overset{‾}{T}}$ the stress tensor, $\overset{‾}{\overset{‾}{S}}$ the strain tensor and c the elasticity tensor. The latter equations are available in the basic version of COMSOL within the *Solid Mechanics* physics module. Extension to the motion of piezo-electrics is classically performed by adding a constitutive electrostatics equation cross-coupled with Hooke’s law, here in stress/charge form:

(9) $$\overset{‾}{\overset{‾}{T}}=c^{E}\overset{‾}{\overset{‾}{S}}-e^{t}E,$$

(10) $$D=e\overset{‾}{\overset{‾}{S}}+{∊}^{S}E,$$

where D is the electric displacement field, E=-∇V the electric field. The system is closed by solving Gauss law ∇·D=0 in the piezo-electrics. The latter equations are available in COMSOL additional *Acoustics module*: extension of Hooke’s law for piezo-electrics is available under an optional sub-node of the *Solid Mechanics* physics module, Gauss law is implemented in a *Electrostatics* physics module, and both modules are coupled through a *Piezoelectric Effect* multi-physics node, implementing Eqs. (9), (10). The *Frequency domain* solver computes the complex displacement field $U_{S}$ in all materials plus complex electric potential *V* in the piezoelectric material. The electric displacement vector field D can further be deduced from Eq. (10) and the input current at the junction between two piezo-electric rings can be computed as:

(11) $$I=\int \int_{S}i\omega \left(D_{2}-D_{1}\right).n_{12}\;\mathrm{dS},$$

where $n_{12}$ is the normal unit vector pointing from piezoelectric disc 1 towards piezoelectric disc 2.

Isotropic losses in materials are considered, in order to account for mechanical energy dissipated in the transducer. A simple way is to use a complex Young modulus

(12) $$E_{\mathrm{eff}}=E_{0}\left(1+i\tan\delta \right)\text{.}$$

Preliminary simulations showed that dissipation occurs mainly in the titanium mass and counter-mass, and in the steel pre-stress screw. Piezoelectric rings would also suffer losses by various mechanical, electrical or electro-mechanical mechanisms, but their contribution to the power dissipated was neglected in this study.

The mechanical boundary conditions on all solid parts exposed to air are free conditions ($\overset{‾}{\overset{‾}{T}}\;n=0$). For the electric boundary conditions of piezo-electrics, the lateral sides are assumed free of surface charge (D.n=0), the faces in contact with mass and counter-mass are grounded (V=0). The classical boundary condition on the interface between the two piezo-electric rings would be an imposed known complex voltage. Here however, to mimic the generator behaviour, we need to impose the input current I. To do so, the boundary between the piezo-electrics is assigned to a voltage *V*, whose value is defined as the solution of a global equation constraining the complex input current *I*, computed from Eq (11), to the required value $I_{0,\mathrm{RMS}}\sqrt{2}$. Finally, Fluid/Structure Interface Conditions must be specified at boundaries separating vibrating solids and fluid (labelled as FSIC on Fig. 3b). These conditions classically express continuity of velocity and stress at the boundary [73], [31] and are easily implementable in COMSOL by an *Acoustics Structure Boundary* multi-physics node.

The complex electrical impedance of the transducer $Z_{\mathrm{Trans}}=V/I$ can be easily computed in any conditions. The impedance curve of the unloaded transducer can be obtained by performing a frequency sweep in a range around the resonance frequency. Since the mechanical properties of metals can slightly vary between different samples, they were fitted by using the experimental impedance curve of the unloaded transducer: the Young modulus of titanium was adjusted so that the computed unloaded resonance frequency matched the experimental one, and its loss angle tanδ so that the computed and experimental impedance module $\left|Z_{\mathrm{Trans}}\right|$ at unloaded resonance matched. The values found by this method (Table 3) are in agreement with commonly reported values [87]. The loss factor for the steel pre-stress screw is set arbitrarily to $\tan\delta =5\times 10^{-4}$, a value representative of results reported in the literature [88]. This procedure ensures that the simulated transducer shows exactly the same unloaded impedance curve as the one used in experiments.

**Table 3:** Mechanical properties of the materials. See text and Refs. [87], [88] for details.

Material	ρ (kg m^−3^)	*E* (GPa)	ν (–)	tanδ (–)
Titanium alloy (TA6V)	4422.43	107.1	0.33	9 × 10^−4^
Steel	7900	196	0.3	5 × 10^−4^
Borosilicate glass	2230	64	0.2	–

### 3.3 Modelling of the control loop

Since the behavior of the piezoelectric transducer is accounted for in the model described above, the input parameters for a given experiments are the same as the experimental ones: frequency *f* and driving current amplitude $I_{0,\mathrm{RMS}}$. However, as described in Section 2.3, frequency is not known by advance and is adjusted by the generator to cancel the imaginary part of the motional branch impedance $IZ_{\mathrm{BM}}=0$. The working frequency is thus load-dependent and this frequency selection method must be reproduced to perform simulations as close as possible to the experimental conditions.

To do so, the static capacity $C_{b}$, which is load independent, is first deduced once for all by a fit of the unloaded impedance curve. Then, for a given configuration ($f,I_{0,\mathrm{RMS}}$, geometry of the liquid), the electrical impedance can be calculated and, knowing $C_{b}$, the motional impedance $Z_{\mathrm{BM}}$ can be deduced by:

(13) $$\frac{1}{Z_{\mathrm{BM}}}=\frac{1}{Z_{\mathrm{Trans}}}-i\omega C_{b}\text{.}$$

We thus proceed as follows: for each configuration, several simulations are performed for a range of step-wise increasing frequencies, close to, and slightly below the resonance frequency. All quantities of interest, especially $Z_{\mathrm{BM}}$, are computed each time. The frequency sought is the one for which $IZ_{\mathrm{BM}}$ crosses zero and is determined by inverse interpolation on the $\left(f,\;IZ_{\mathrm{BM}}\right)$ curve. This allows an exact mimicking of the generator strategy, at the price of performing multiple simulations for a given configuration. More details and illustrations can be found in Ref. [57].

## 4 Results

### 4.1 Variable current

**Fig. 4:**
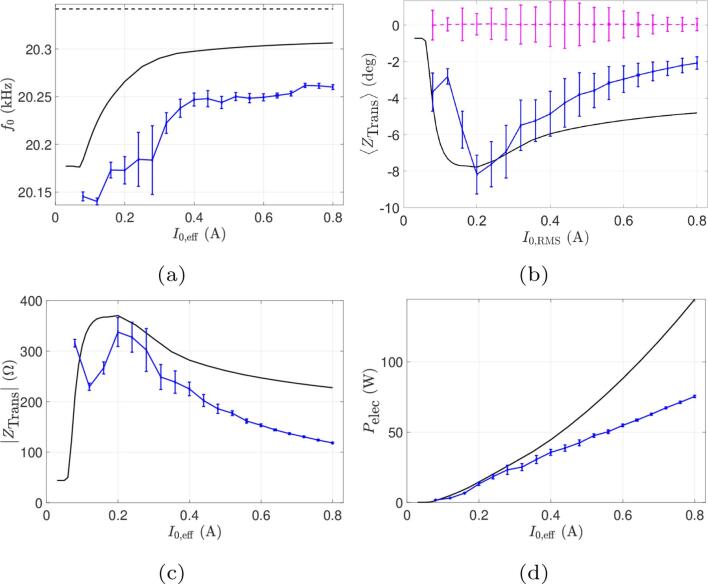
Comparison between simulations and experiments for beaker A at constant immersion depth and varied current. Continuous curves (black lines) represent numerical results and curves with error bars (blue online) represent experimental results. (a) Operating frequency. (b) Phase of transducer electrical impedance. The thin line (pink online) is the measured phase of the motional impedance. (c) Module of transducer electrical impedance. (d) Electrical power consumed.

In this set of experiments, the liquid geometry is kept fixed and the input current amplitude is varied. The comparison between simulations and experimental results is shown in Fig. 4. The operating frequency of the system chosen by the generator is displayed in Fig. 4a: it can be seen that the simulation correctly captures the frequency evolution with current, which asymptotes to a value about 30Hz below the unloaded resonance frequency, indicating that our model produces a reasonable estimate of the load seen by the transducer despite a slight difference of approximately 50Hz between numerical and experimental results (which is less than 0.25% error).

The phase of the transducer impedance is shown in Fig. 4b. The numerical curve reasonably reproduces the experimental result, with a minimum value near 0.2 A. The descending part of the curve corresponds to a transition from a constant phase (leftmost part of the curve), when there is no cavitation and linear acoustics holds, up to the progressive buildup of a cavitation cloud at the transducer tip. Fig. (4b) also display the experimental and computed phase of the motional branch impedance. Apart from small fluctuations of about 1°, $Z_{\mathrm{BM}}$ is close to zero, which clearly demonstrates that the generator correctly ensures a purely real impedance of the motional branch.

Fig. 4c shows that the shape of the transducer impedance modulus vs. current is also correctly predicted by the model, except for very low currents. So far, we don’t have a clear explanation for this mismatch, but within this range of low currents, the acoustic impedance seen by the transducer varies considerably, rendering frequency control more difficult. Furthermore, the generator does not allow the use of very low input currents, making a further comparison in this area difficult. Finally, in Fig. 4d, the computed and experimental electrical power consumed are compared. Our simulation, as can be seen, overestimates the electrical power by a factor of about two. Furthermore, the experimental curve is almost linear with increasing currents, whereas the numerical curve bends upwards. This suggests that our model overestimates the energy dissipated by cavitation in the liquid.

**Fig. 5:**
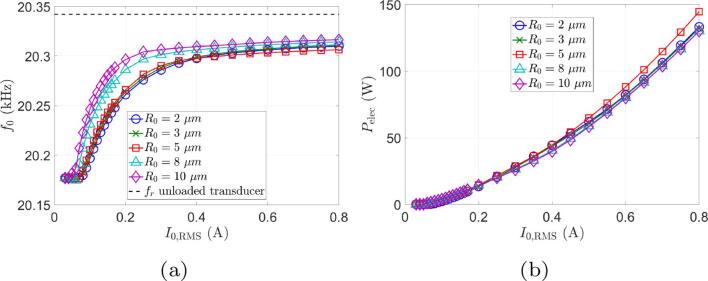
Sensitivity of results to the bubble radius $R_{0}$. The bubble density is set to 50bubbles/mm^3^. (a) Operating frequency. (b) Electrical power consumed.

**Fig. 6:**
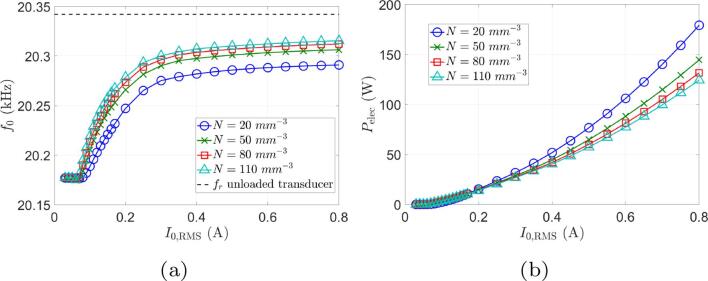
Sensitivity of results to the bubble density *N*. The ambient radius is $R_{0}=5\mu \text{m}$. (a) Operating frequency. (b) Electrical power consumed.

In order to examine the sensitivity of the results to the model free parameters, computations were repeated for $R_{0}$ in the range 2μm to 10μm and $N_{0}$ in 20bubbles/mm^3^ to 110bubbles/mm^3^. The same conclusions can be drawn, apart from very slight changes in the other output variables, as shown in Fig. 5, Fig. 6, respectively.

### 4.2 Variable transducer immersion

The influence of the beaker geometry and the immersion depth of the transducer were investigated. The three different-shaped beakers formerly labeled as A, B, and C were used in experiments for various transducer immersion conditions, and the corresponding acoustic fields were computed with our model. The input current of the transducer was set to 0.6 A for all experiments.

**Fig. 7:**
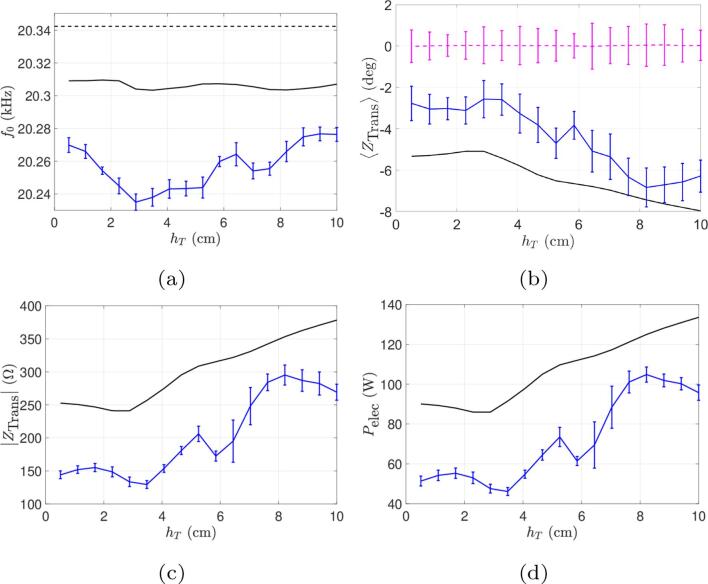
Comparison between simulations and experiments for the beaker A for the experience at a constant current ($I_{\mathrm{eff}}=0.6\;A$) and varied immersion depth. Continuous curves (black online) represent numerical results and curves with error bars (blue online) represent experimental results. (a) Operating frequency. (b) Phase of transducer impedance. The horizontal dotted line (pink online) is the measured phase of the motional impedance (constrained to zero in computations). (c) Module of transducer electrical impedance. (d) Electrical power consumed.

**Fig. 9:**
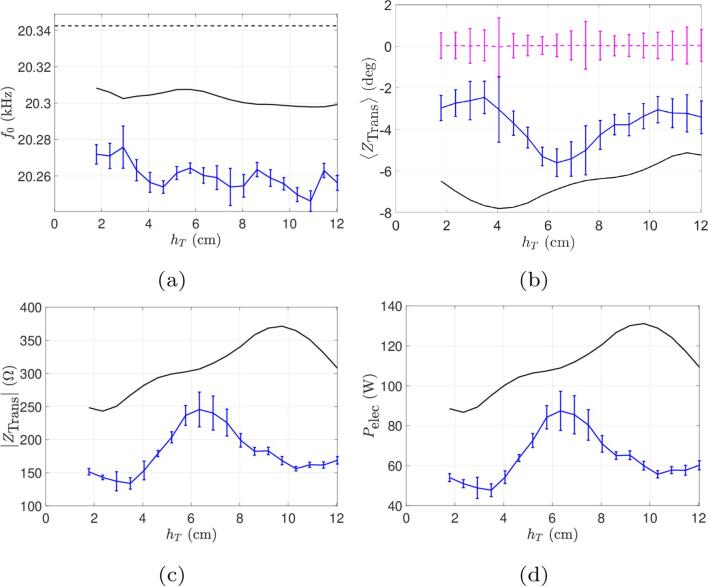
Comparison between simulations and experiments for the beaker B for the experience at a constant current ($I_{\mathrm{eff}}=0.6\;A$) and varied immersion depth. Continuous curves (black online) represent numerical results and curves with error bars (blue online) represent experimental results. (a) Operating frequency. (b) Phase of transducer impedance. The horizontal dotted line (pink online) is the measured phase of the motional impedance (constrained to zero in computations). (c) Module of transducer electrical impedance. (d) Electrical power consumed.

**Fig. 10:**
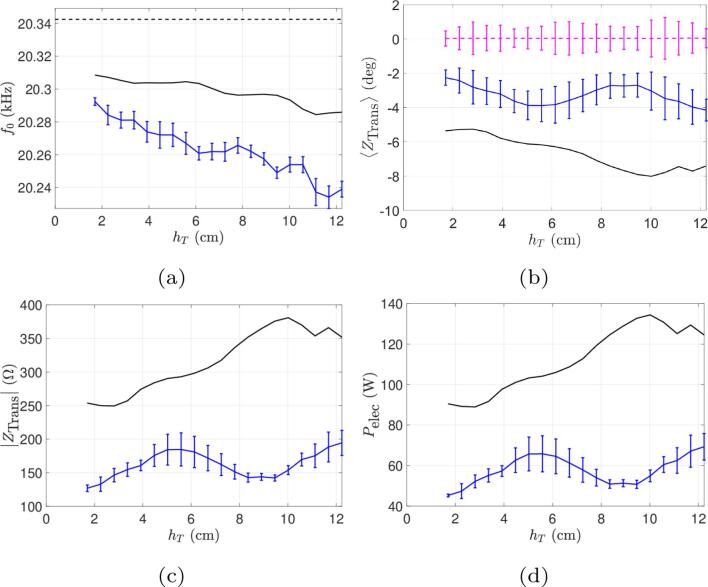
Comparison between simulations and experiments for the beaker C for the experience at a constant current ($I_{\mathrm{eff}}=0.6\;A$) and varied immersion depth. Continuous curves (black online) represent numerical results and curves with error bars (blue online) represent experimental results. (a) Operating frequency. (b) Phase of transducer impedance. The horizontal dotted line (pink online) is the measured phase of the motional impedance (constrained to zero in computations). (c) Module of transducer electrical impedance. (d) Electrical power consumed.

The comparisons between numerical results and experimental data for the main electrical output quantities in function of immersion depth $h_{T}$ are shown in Fig. 7, Fig. 9, Fig. 10, for beakers A, B and C, respectively.

**Fig. 8:**
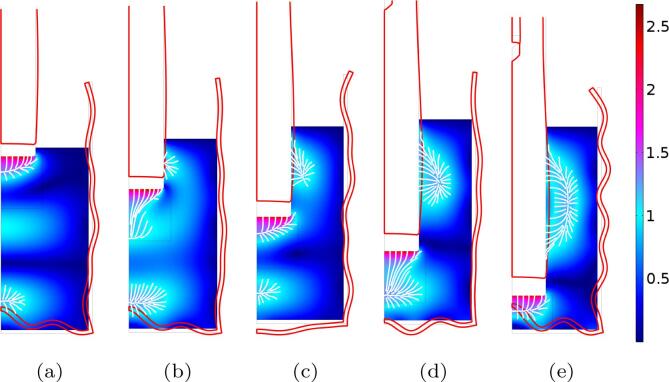
Computed acoustic fields for beaker A (Fig. 7), for different immersion depths (from left to right: $h_{T}=0.52,2.89$, 5.26,7.63,10cm). The color-plot represents the acoustic field amplitude in bar. The white lines are the computed paths of bubbles in regions above the Blake threshold, assuming they follow the primary Bjerknes force field [60]. The red line are the deformed shape of the transducer and beaker’s walls.

For the 1L beaker A, Fig. 7e shows that the electrical power increases with immersion depth, i.e. as the tip of the transducer approaches the bottom of the vessel. Our model captures this feature reasonably well, and this reflects the progressive enlargement of the cavitation zone on the lateral sides of the transducer as immersion increases. This is evidenced on Fig. 8 which shows the acoustic field and the cavitation zones for various immersion depths. This increase of dissipated power with immersion is a general trend in such experimental configurations [89], but other acoustic resonance effects may qualify this conclusion. For example, the peaks observed for some immersion depths in the power curve of beaker A on Fig. 7e are not captured by our simulations. The situation becomes more complex for larger volumes, as will be seen for beakers B and C. On the other hand, it can also be seen on Fig. 7e that here again, our computations overestimate the active electrical power by a factor of about two. This conclusion also holds for beakers B and C (Fig. 9, Fig. 10d).

The experimental and computed values of the phases of transducer and motional branch impedances are superimposed on Figs. 7b, 9b and 10b for the three beakers. It can be seen that in all cases, the generator correctly maintains $IZ_{\mathrm{BM}}=0$ despite the variations of the load as immersion depth varies (pink dashed line), which demonstrate that the control-loop for automatic frequency locking works properly. On the other hand, for beaker A (Fig. 7c), our model reasonably captures the variations of $IZ_{\mathrm{Trans}}$, and our estimation of the working frequency differs from the experimental one by less than 100Hz (Fig. 7a).

It is interesting to compare Fig. 7, Fig. 7, which show that the variations of the impedance module $\left|Z_{\mathrm{Trans}}\right|$ closely follows the ones of the dissipated power, both experimentally and in computations. This is a natural result since the phase of $Z_{\mathrm{Trans}}$ is close to zero so that $\left|Z_{\mathrm{Trans}}\right|$ $≃R(Z_{\mathrm{Trans}})$. Since the electrical power reads $P_{\mathrm{elec}}$ =  $R(Z_{\mathrm{Trans}})I_{0,\mathrm{RMS}}^{2}$, both curves are therefore proportional as long as the input current remains constant. Our overestimation of $P_{\mathrm{elec}}$ is therefore also naturally reflected on $\left|Z_{\mathrm{Trans}}\right|$.

A practical instructive conclusion can be drawn from the power curves in function of immersion depth. The imposed current value corresponds in fact to a given position of the “power level” button on the generator. It can be seen therefore that fixing this position does not ensure a given active power since as shown on Fig. 7, Fig. 9, Fig. 10d the latter is immersion depth-dependent. The case of beaker B is particularly interesting since the dissipated power exhibits a marked maximum for an immersion depth slightly above 6 cm and a minimum near 10 cm. Thus, since the liquid volume remains constant, two experimenters working with the same graduation level, but with these different immersion depths, would conduct sonochemistry experiments with very different dissipated powers.

Going further into this issue, Fig. 9, Fig. 10d show that our model also yields extrema in the power curves of beakers B and C, but not at the same locations as for the experimental ones. We suspect that the presence of these extrema reflects some acoustic resonance effects of the vessel for specific immersion depths. Were the liquid governed by linear acoustics, the location of these peaks would be independent of the driving amplitude $I_{0,\mathrm{RMS}}$. But as the liquid behaviour is nonlinear, their location may vary with amplitude. As shown so far, our model overestimates the energy dissipated in the liquid, possibly predicting cavitation zones larger than the experimental ones, and therefore more non-linearity. The discrepancy between computed and experimental results observed for the locations of the power extrema might therefore be a further consequence of our overestimation of the dissipated power.

**Fig. 11:**
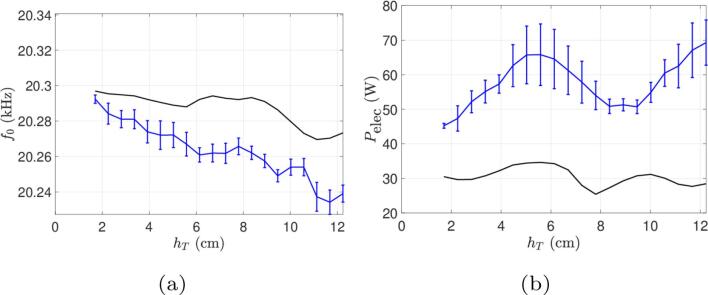
Comparison between simulations and experiments for the beaker C for the experience at a constant current and varied immersion depth. Continuous curves (black lines) represent numerical results at $I_{\mathrm{eff}}=0.3\;A$ and curves with error bars (blue lines) represent experimental results at $I_{\mathrm{eff}}=0.6\;A$. (a) Operating frequency and (b) Electrical power consumed.

In order to check whether the correct peak locations can be recovered for smaller drivings, the current amplitude was lowered to $I_{\mathrm{eff}}=0.3\;A$ in the simulations of beaker C, and compared to the experimental curve at $I_{\mathrm{eff}}=0.6\;A$ (Fig. 11): the shape of the computed power curve now fits much better the experimental one, and exhibit extrema at the same immersion depths, but the electrical power is now underestimated.

The above considerations clearly show that the vessel and liquid geometry noticeably influence the energy transfer from the transducer to the liquid, which is a manifestation of acoustic effects. Our model captures such effects qualitatively, but is yet unable to make quantitatively correct predictions. Similar results have been reported in the literature [90], [91], [92], [93], [94], [95], [89], [96] and deserve further investigations.

## 5 Discussion

The orders of magnitudes and trends of our predictions are in reasonable agreement with experimental results. Owing to the numerous multi-physics aspects of our simulations, which mimic as close as possible a sonoreactor experiment, these results are rather encouraging. But the observed systematic overestimation of the dissipated electrical power by a factor of about two deserves further discussion. As the latter directly reflects the mechanical power dissipated by cavitation, this reflects a general overestimation of the dissipated power by cavitation bubbles in the model of Ref. [59], despite the latter is based on real bubble dynamics. Apart from the semi-arbitrary choice of parameters $R_{0}$ and *N*, whose parametric variations have shown negligible influence, several enhancements should be considered.

One well-known weakness of our model is the arbitrary setting of the real part of $k^{2}$ to its classical linear expression (4), which is independent of pressure amplitude. This issue has been recently re-examined independently by Trujillo [97], [64] and Sojahrood and co-workers [66], [68]. They showed that both real and imaginary parts of $k^{2}$ can be expressed as period-averaged expressions involving the pressure field and the local void fraction $\beta =N4\pi R^{3}/3$ (with possible extension to poly-disperse bubble sizes). The authors finally obtain the same expression for $I(k^{2})$ as Eq. (5) but $R(k^{2})$ is also found to depend on the local acoustic pressure |P|. This not only affects the local sound velocity but also the attenuation. Calculations for uncoated 2μm bubbles showed that the values of $R(k^{2})$ can drastically fall as acoustic pressure increases, in an increasing range of frequencies around resonance [68]. Interestingly, the authors found that the drop of $R(k^{2})$ could yield values of attenuation falling to near 50% of our own estimation, which is the order of magnitude of the discrepancy observed in the present work. Whether similar conclusions could still be drawn for the low $f/f_{\mathrm{res}}$ ratio used in the present study remains to be clarified, and including the correct value of $R(k^{2})$ in our model is part of an ongoing work.

Our model still disregards the radiation losses [63], [64], [67], [65], despite we use the Keller equation in our numerical computations. The historical reason for that is that the Caflisch model, from which our model is a simplification, was rigorously derived with the Rayleigh-Plesset equations. To what extent the latter can be replaced by a compressible bubble dynamics in the fully nonlinear model remains to be explored, despite it is a well-known practice in linear versions [55], [98], [99], [100], [101]. Nevertheless, accounting for the radiative dissipation term has now become common, and it will be included in a future version of our model. One should note however that adding this term would further amplify the discrepancy observed in the present study.

Another possible overestimation of attenuation may be due to the fact that the bubble cloud is very dense in some regions (especially near the transducer), invalidating the basic scaling assumptions of Caflisch model, which is valid to O(β). This should be solved by accounting for bubble–bubble interactions more carefully than the way they are modelled in O(β) models, where bubbles interact only through their individual coupling with the mean field. Some extensions have been explored for example by [98] under the linear assumption, and in a more general context by [44] which yield better agreement with the well-known experimental results of Silberman [102] once linearized. Another approach may consist in solving coupled radial dynamics of a given set of *N* randomly dispersed bubbles [103], [104] extending the two-bubbles model of Ref. [105]. The latter approach also yields better agreement with Silberman’s experiments near resonance [68]. We think however that such subtle refinements would probably compromise the simplicity of our model, whose efficiency is guaranteed by the ability to compute the power dissipated by a single bubble upstream from the COMSOL model (see A).

Bubble size poly-dispersity may also alter the dissipated power evaluation, despite the parametric study proposed in the present paper shows little sensitivity of the global power consumed to the precise value of bubbles ambient radius. The recent study of Lesnik and co-workers [72] shows however some interesting spatial segregation of bubbles depending on their sizes. Bubbles sizes are primarily bounded from above by bubble breakup, and owing to surface tension, small bubbles are expected to be more resilient against large acoustic pressures. Defining a main bubble size dependent on the local acoustic pressure in our model is under investigation.

Finally, we would like to stress another flaw in our model, which is related to the location we choose for those inertial bubbles that dissipate energy, as defined by Eqs ((2c), (2d)). The latter impose the presence of bubbles in any zone where the acoustic pressure lies beyond the Blake threshold. However, it is well-known that in a standing wave, the primary Bjerknes force can become repulsive above some threshold, owing to the increasingly long expansion phase of inertial bubbles with the driving pressure [106], [107], [62], [108], [86], [60]. This threshold is close to 1.75bar in water, and this feature is known to yield the so-called “Jellyfish structures” [109], [110], [111], [62], where a large pressure antinodes in a standing wave zone repels the bubbles toward the threshold location. Eqs. ((2c), (2d)) do not catch this subtlety and blindly puts some bubbles in such regions, which prohibits the prediction of such bubble structures in our simulations. Our model thus predicts streamers that are not present in experiments, and erroneously compute some dissipation there. In an ongoing work extending the present one, we performed comparisons between luminol maps and the model’s predictions, that would tend to support this interpretation. The shift in the predicted locations of the extrema on Fig. 9, Fig. 10d might also stem from the same issue. Whereas the other above-mentioned imperfections of our model lied in the potential inaccuracy in the evaluation of the bubble dissipated power, the present one is due to the incorrect prediction of the spatial location of bubbles. Correcting this problem rigorously would thus require modelling the translational motion of bubbles as in Ref. [72]. However, in order to keep reasonable computation costs, we plan to use an in-between solution in order to patch the condition used in Eqs. ((2c), (2d)), taking advantage of the ability of our model to compute the primary Bjerknes force field [60].

## 6 Conclusions

Louisnard’s simplified model of nonlinear sound propagation in cavitating liquids [59] has been included in a global sonoreactor model, accounting for the vessel walls vibrations, the transducer, and the automatic frequency control strategy of the generator. For a given geometrical configuration, the only required parameter of the simulation is the amplitude of the input current, which is the quantity imposed by the power button of the generator. Therefore, our simulations exactly mimic experiments, without resorting to debatable arbitrary boundary conditions. Our model provides estimates of experimentally measurable quantities such as working frequency, electrical complex impedance, and consumed electrical power. To the best of our knowledge, this is the first reported model allowing such fine and comprehensive simulations of acoustic cavitation experiments, allowing large parametric studies, and directly testable against experimental data.

Experiments were also performed, varying either the input current or the transducer immersion depth, for three different beaker geometries. Our global model was put to test against the electrical data provided by the ultrasound generator in the latter experiments. Despite the correct order of magnitudes for frequency, impedance and electrical power are caught by our simulations, there emerges a net tendency of our model to overestimate the consumed electrical power, by a factor of about two. Parametric variations of the bubble ambient radius and bubble density in our model did not fix the discrepancy observed.

Geometrical effects can be clearly observed both in experiments and simulation, and materialize as extrema in the consumed electrical power when the transducer immersion depth is varied, but some discrepancy is observed on the location of these extrema. This suggests that strong variations of a sonochemical yield may be observed when changing the transducer immersion depth at constant input current and constant liquid volume. Understanding and correctly predicting these effects to optimize the shape of sonoreactors is a future challenge for extensions of the present model.

The discrepancies observed between theory and experiments open the way to future enhancements of Louisnard’s model. Moreover, ongoing experimental work extending the present study, including luminol and quantitative sonochemistry, is currently under investigation.

## CRediT authorship contribution statement

**Igor Garcia-Vargas:** Software, Investigation, Data curation, Writing - original draft. **Laurie Barthe:** Conceptualization, Methodology, Validation, Resources, Project administration, Funding acquisition. **Pascal Tierce:** Validation, Resources, Supervision, Project administration, Funding acquisition. **Olivier Louisnard:** Conceptualization, Methodology, Software, Investigation, Data curation, Writing - review & editing, Funding acquisition.

## Declaration of Competing Interest

The authors declare the following financial interests/personal relationships which may be considered as potential competing interests: Olivier LOUISNARD reports financial support was provided by SinapTec. Laurie BARTHE reports financial support was provided by SinapTec. Igor GARCIA-VARGAS reports a relationship with SinapTec that includes: employment and funding grants.

## Appendix A Bubble dynamics model

The dynamics of the bubbles is computed by a Keller equation, accounting to the liquid compressibility up to first order in Mach number $\overset{˙}{R}/c_{l}$ [112], [113]:

(A.1) $$\begin{array}{cc} & R\frac{d^{2}R}{{\mathrm{dt}}^{2}}\left(1-\frac{1}{c_{l}}\frac{\mathrm{dR}}{\mathrm{dt}}\right)+\frac{3}{2}{\left(\frac{\mathrm{dR}}{\mathrm{dt}}\right)}^{2}\left(1-\frac{1}{3c_{l}}\frac{\mathrm{dR}}{\mathrm{dt}}\right)=\\ & \;\frac{1}{\rho_{l}}\left[\left(1+\frac{1}{c_{l}}\frac{\mathrm{dR}}{\mathrm{dt}}\right)\left(p_{g}-p_{\infty }(t)\right)+\frac{R}{c_{l}}\frac{{\mathrm{dp}}_{g}}{\mathrm{dt}}\right)\\ & \;\left(-\frac{2\sigma }{R}-4\mu_{l}\frac{1}{R}\frac{\mathrm{dR}}{\mathrm{dt}}\right] \end{array}$$

with R(t) is the bubble radius, $c_{l},\rho_{l}$ and $\mu_{l}$ are the sound velocity, density and viscosity of the liquid, respectively, $p_{g}$ is the pressure in the bubble, σ the surface tension, and

(A.2) $$p_{\infty }(t)=p_{0}(1-P^{*}\sin2\pi \mathrm{ft})$$

is the sinusoidal driving pressure at frequency *f*, around the ambient pressure $p_{0}$.

We take this opportunity to report an error in our original paper [59]: we reported there that we simulated the Rayleigh equation in order to obtain the dissipated power $\Pi_{v}$ and $\Pi_{\mathrm{th}}$. In fact the Keller equation was used and this is true in all our subsequent work, including the present one.

The pressure in the gas is assumed uniform. The gas content is a mixture of several species (here $H_{2}O$ and air), with number of moles $n_{i}$, represented by an approximate van der Waals equation of state:

(A.3) $$p_{g}=\frac{\underset{i}{\sum }n_{i}RT}{4/3\pi R^{3}-b},$$

where R is the universal gas constant. Following [114], the reduced volume is computed as averages of the individual species reduced volumes:

(A.4) $$b=\frac{\underset{i}{\sum }n_{i}b_{i}}{\underset{i}{\sum }n_{i}}$$

To account for the bubble thermal behaviour and heat transfer with the liquid we followed the following simplified approach: the bubble internal temperature is assumed uniform except in a near-wall layer of thickness $\delta_{\mathrm{th}}$ [115], [116], [117]. The latter is estimated by:

(A.5) $$\frac{\delta_{\mathrm{th}}}{R(t)}=\min\left({\mathrm{Pe}}_{\mathrm{th}}^{-1/2},1/\pi \right),$$

where ${\mathrm{Pe}}_{\mathrm{th}}=R\overset{˙}{R}/\alpha_{g}$ is a thermal Peclet number, $\alpha_{g}$ is the thermal diffusivity of the gas mixture in the bubble. The cutoff 1/π proposed in Ref. [115] is needed to avoid nonphysical large values of $\delta_{\mathrm{th}}$ during nearly isothermal phases of the bubble oscillation. It was shown by Preston et al. [117] that the correct value of the cutoff is rather 1/5.

Following this approach, the temperature of the bubble core can be obtained by applying the first law of thermodynamics to the whole bubble content:

(A.6) $$\frac{\mathrm{dU}}{\mathrm{dt}}=-p_{g}\frac{d(4/3\pi R^{3})}{\mathrm{dt}}+{\overset{˙}{n}}_{H_{2}O}h_{H_{2}O}+\lambda \frac{T_{l}-T}{\delta_{\mathrm{th}}}\text{.}$$

In the latter equation, the left-hand side is the time-derivative of the bubble gas mixture internal energy

(A.7) $$U=\underset{i}{\sum }n_{i}\int_{T_{0}}^{T}C_{v_{i}}(T\prime )\mathrm{dT}\prime ,$$

the first term in the right-hand side of (A.6) is the rate of work done by the liquid on the bubble, the second is the enthalpy flux of water evaporating from (or condensing into) the liquid with a molar flux ${\overset{˙}{n}}_{H_{2}O}$, and the last one is the conduction heat transfer between the bubble and the liquid, assumed at constant undisturbed temperature from ambient [115].

To account for water evaporation/ condensation at the bubble wall, an approach similar to heat transfer is used. Water vapour concentration is assumed constant in the bubble except in a near-wall layer of thickness

(A.8) $$\frac{\delta_{m}}{R(t)}=\min\left({\mathrm{Pe}}_{m}^{-1/2},1/\pi \right),$$

which acts as a barrier to diffusive transfer between the bubble center and the wall [118], [116]. In Eq. (A.8), ${\mathrm{Pe}}_{m}=R\overset{˙}{R}/D_{g,H_{2}O}$ is the mass Peclet number, where $D_{g,H_{2}O}$ is the diffusion coefficient of water and the mixture of gases present in the bubble.

The conservation equation of water reads therefore:

(A.9) $$\frac{{\mathrm{dn}}_{H_{2}O}}{\mathrm{dt}}=4\pi R^{2}D_{g,H_{2}O}\frac{C_{H_{2}O}(R)-C_{H_{2}O}}{\delta_{m}},$$

where

(A.10) $$C_{H_{2}O}(R)=\frac{p_{\mathrm{sat}}(T_{l})}{RT_{l}},\;C_{H_{2}O}=\frac{n_{H_{2}O}}{4/3\pi R^{3}}$$

are the water vapour concentrations at the bubble wall and in the bubble center, respectively. The molar flux ${\overset{˙}{n}}_{H_{2}O}$ in Eq. (A.6) is equal to the right-hand-side of Eq. (A.9).

The $C_{v}(T)$ and $C_{p}(T)$ are computed from Ref. [119]. Following [120], the gas mixture conductivity is computed using method of Chung et al. see [121] Sections 10.3 and 9.5. This may allow estimations of λ for high density states, but due to convergence issues, we followed Ref. [115] and estimated λ at liquid temperature. We also followed Storey & Szeri [120] to compute the diffusion coefficient between air and water, with the Brokaw correction to account for polar molecules (here water) see [121] Eq.  (11.3.7) and followings. Despite the correction proposed in appendix of Ref. [120] to account for high densities is available in our code, the diffusion coefficient was also estimated at liquid temperature.

The set of differential equations (A.1), (A.6) and (A.9) is solved with a FORTRAN code using the stiff solver DDRIV3 from NIST Core math library (CMLIB), over 200 acoustic periods. The solution yields the unknowns R(t),T(t) and $n_{H_{2}O}(t)$, from which the gas pressure can be obtained from Eq. (A.3). The power dissipated by the bubble over the last cycle can then be estimated with the integrals:

(A.11) $$\Pi_{\mathrm{th}}=\frac{1}{T}\int_{0}^{T}-p_{g}\frac{\partial V}{\partial t}\;\mathrm{dt},$$

(A.12) $$\Pi_{v}=\frac{1}{T}\int_{0}^{T}16\pi \mu_{l}R{\left(\frac{\partial R}{\partial t}\right)}^{2}\;\mathrm{dt}\text{.}$$

The graphs reported in our original paper [59] were obtained with the above model and $\Pi_{\mathrm{th}}$ was found to be much smaller than $\Pi_{v}$ in the parametric range of interest, which justifies that only the latter is retained in Eq. (5). It should be noted that a different conclusion was found by Jamshidi & Brenner [63], [72]. This might be due to the fact that the latter authors cut the computation of the integral at the end of the collapse, rather than over a whole bubble cycle. Contrarily to the integrand in the viscous term in (A.12) which is always positive, there are sign changes in the one of (A.11), as the heat flow changes direction along the oscillations (even if the integral over one cycle must be positive). The latter authors would miss therefore some post-collapse events involving an inward heat flow occurring during part of the rebounds, associated with very low transient temperatures attained in the bubble, consecutive to rapid adiabatic re-expansions [122]. Other reasons for discrepancy may lie in the tricky computation of thermal properties of the gas mixture.

The bubble response is computed by parametrically varying the dimensionless driving amplitude $P^{*}$ by small steps between 0 and 3, to compute the viscous contribution $\Pi_{v}$, for a given ambient radius $R_{0}$ and frequency *f*. The resulting curve can be perfectly fitted in dimensionless form by:

(A.13) $$\frac{\Pi_{v}/p_{0}V_{0}\omega }{{P^{*}}^{2}}=\exp\left[K_{1}+\frac{2}{\pi }K_{2}\tan^{-1}\left(K_{3}P^{*}-K_{4}\right)\right],$$

and is injected in COMSOL as an explicit function to compute $I(k^{2})$ from (5).

We emphasize that the whole procedure must be repeated for any change of $R_{0},f$, as well as when changing the liquid or the bubble gas content, or the ambient conditions. A universal analytical expression for $\Pi_{v}$ avoiding this straightforward but lengthy procedure is currently under study.
